# Differential concentrations of NaCl and K_2_Cr_2_O_7_ stress conditions: biophoton characteristics and quality prediction feasibility analysis of fresh *Nepeta cataria* L. leaves

**DOI:** 10.3389/fpls.2025.1714452

**Published:** 2025-11-12

**Authors:** Ruyu Wang, Jiahui Luan, Zhiying Wang, Baorui Cao, Yiwei Chen, Xue Li, Tingting Deng, Jinxin Du, Jinxiang Han, Meina Yang

**Affiliations:** 1 Department of Clinical Pharmacy, The First Affiliated Hospital of Shandong First Medical University & Shandong Provincial Qianfoshan Hospital, Shandong Medicine and Health Key Laboratory of Clinical Pharmacy, Jinan, China; 2 Biomedical Sciences College & Shandong Medical Biotechnology Research Center, National Health Commission Key Laboratory of Biotechnology Drugs, Shandong First Medical University & Shandong Academy of Medical Sciences, Jinan, China; 3 College of Traditional Chinese Medicine, Shandong University of Traditional Chinese Medicine, Jinan, China; 4 Shandong Women’s University, School of Healthy Aging, Jinan, Shandong, China

**Keywords:** NcL, biophoton, SPE, DL, CPS, I_0_

## Abstract

**Introduction:**

*Nepeta cataria* L. (NcL), a perennial medicinal plant, is used to dispel wind-heat, remove blood stasis, and reduce swelling. However, soil salinization and heavy metal pollution have severely impacted its growth and quality. Current quality assessments, based solely on pulegone (PG) content in the final product, neglect cultivation process controls.

**Methods:**

We applied biophoton detection to differentiate stress types in fresh NcL leaves and explored the correlation between biophoton emission and plant quality. Spontaneous photon emission (SPE) and delayed luminescence (DL) were measured under NaCl and K_2_Cr_2_O_7_ stress. Key parameters, including counts per second (CPS) and initial intensity (I_0_), were analyzed. PG content was determined via ultra-high performance liquid chromatography, alongside physiological and oxidative stress indexes.

**Results:**

Stress conditions significantly inhibited NcL growth and quality, leading to marked differences in biophoton emission, physiological and biochemical indicators, oxidative stress indexes and PG content between stress and control groups. For instance, in the 120 mmol/L NaCl group, the CPS decreased by 54.27%, while I_0_ dropped by 54.12%. PG content in high-salinity groups was 1.22 mg/g, 40.19% lower than controls (2.04 mg/g). CPS and I_0_ strongly correlated with PG content (r = 0.89 and r = 0.85, respectively), as well as with physiological and oxidative indicators. These relationships were clearly visualized.

**Discussions:**

Our study demonstrates the potential of biophoton detection for quality evaluation during NcL cultivation. We identified specific biophoton parameters that reflect plant quality, offering a new approach for process-based quality control.

## 1 Introduction

*Nepeta cataria* L. (NcL) is a perennial herb widely recognized in traditional medicine across China and Europe for its properties in dispelling wind-heat, removing blood stasis, and reducing swelling (Sharma et al., 2021; Board, 1975; Chinese Materia Medica Editorial Board, 1999; Adiguzel et al., 2009; Borlace et al., 2024; Fan et al., 2017; Nadeem et al., 2022; Suntar et al., 2018; Tan et al., 2019; Tiwari et al., 2023). Its chemical composition is dominated by volatile oils, flavonoids, terpenoids, phenolic compounds, and an array of trace constituents (Heuskin et al., 2009; Modnicki et al., 2007; Wang et al., 2007). Its bioactivity is largely attributed to volatile oils, with PG and menthone being the principal components and key markers for quality control (Flamini et al., 1999; Sarheed et al., 2020; Shahdadi et al., 2023; Zhao et al., 2022; Suzhen, 2024).

The quality and medicinal value of NcL are profoundly determined by environmental factors from its source. Furthermore, the growth and accumulation of bioactive compounds in NcL, such as PG, are significantly influenced by environmental factors, including light intensity, soil conditions (e.g., salinity and pH), and exposure to pollutants like heavy metals (Yafang et al., 2023; Karami et al., 2022; Lungoci et al., 2023; Xu et al., 2024). While agronomic practices can be optimized to some extent (Gomes et al., 2024; Duppong et al., 2004), a critical gap remains in the ability to perform rapid, non-destructive, and *in vivo* assessment of plant physiological status and metabolic quality in real time. Current methods often rely on destructive sampling and ex post facto chemical analysis, which hinder dynamic monitoring and early stress detection. This limitation underscores the need for innovative approaches, such as biophotonic technology, to directly evaluate plant stress responses and predict secondary metabolite content without compromising plant integrity.

Current quality control of NcL, as stipulated by the Chinese Pharmacopoeia, primarily relies on quantifying PG in the final dried product using techniques like HPLC and GC-MS (Commission., 2020; Xingchen et al., 2023; Suzhen, 2024; Dongjing et al., 2010; Jingbo et al., 2014; Yunyun et al., 2014; Yanwu et al., 2020). While essential, these methods are post-harvest, destructive, and detached from the dynamic growth process. They cannot provide real-time feedback on plant health or the dynamic formation of quality during cultivation. Therefore, a paradigm shift towards non-destructive, process-oriented quality monitoring is necessary to ensure consistent herbal quality.

Biophoton emission, also known as ultra-weak photon emission (UPE), is characterized by the spontaneous and continuous emission of broad-spectrum photons from living organisms under metabolic activity, requiring highly sensitive devices for detection due to its low intensity (Cohen and Popp, 1997; Du et al., 2023; Havaux et al., 2006). The generation of biophotons is initiated by the transition of molecules from high-energy to low-energy states, a process that carries information reflective of the organism’s microscopic physiological status (F. Hua, 2016).

The primary motivation for employing biophoton emission as a stress indicator lies in its direct link to oxidative metabolism. This connection is central to the “metabolic-luminescence” theory, which posits that UPE originates from electronically excited species formed during oxidative metabolic processes and stress, particularly those involving reactive oxygen species (ROS) and lipid peroxidation (Cifra and Pospisil, 2014; Shuli et al., 2004). This mechanistic rationale positions biophotonic parameters as potent, non-invasive indicators of internal oxidative status.

Biophoton emission is categorized into two types: SPE, an inherent, continuous low-intensity glow, and DL, which is a transient emission with hyperbolic decay following an external light stimulus. The behaviors of SPE and DL are closely related to the sample’s intrinsic properties, revealing its current state and internal structure. An alternative perspective is offered by the “coherent radiation” theory, which posits that biophotons emerge from highly coherent electromagnetic fields within the biological system, reflecting its totality (Popp et al., 1988; Jinxiang and Yi, 2011; Qiao, 1988). The spectral characteristics of UPE, typically between 450–700 nm for metabolic origins, further underpin its analytical value (Ge et al., 2006). The established link to oxidative stress mechanisms underscores the significant potential of biophoton technology for the non-destructive monitoring of cultivation stress and the quality evaluation of medicinal plants.

Preliminary research has demonstrated the promise of biophoton technology in Traditional Chinese Medicine (TCM) quality assessment. Studies have successfully correlated biophoton parameters (SPE and DL) with the growth age, variety, and content of active ingredients in herbs like *Platycodon grandiflorus* (Jacq.) A.DC., *Salvia miltiorrhiza* Bge., and *Lonicera japonica* Thunb. (X. Zhao et al., 2017). Furthermore, DL has been used to differentiate batches of ginsenoside extracts, monitor quality dynamics in *Carthamus tinctorius* L., and shows high potential for authenticating TCM due to its sensitivity to compositional changes and its consistency with chemical analysis outcomes (Sun et al., 2018, 2019; Cao et al., 2023). Collectively, these findings establish biophoton emission as a holistic and non-destructive indicator of plant physiological and metabolic status.

However, the application of this technology specifically to the quality evaluation of fresh NcL under abiotic stress remains unexplored. This study aims to bridge this gap. We established NaCl and K_2_Cr_2_O_7_ stress models to systematically investigate the biophoton characteristics (SPE and DL) of fresh NcL leaves. Our objectives are to: 1) clarify the changing patterns of biophoton parameters under different stress conditions; 2) investigate the correlation between these parameters and key indicators, including PG content, physiological and biochemical indicators, and oxidative stress indexes; and 3) identify reliable biophoton characteristic parameters for distinguishing NcL of different qualities, thereby validating the feasibility of biophoton technology for the non-destructive prediction of NcL quality during cultivation.

## 2 Materials and methods

### 2.1 Herbal cultivation and stress treatments

#### 2.1.1 Herbal cultivation

##### 2.1.1.1 Seed soaking

Prior to sowing, it is imperative that the seeds be pre-treated. The following procedure was employed: the seeds were placed in a beaker and warm boiled water at 50-60°C was slowly poured in, stirring as it was poured, for about 10 min. Subsequently, the seeds were immersed in the water for a period of 24 hours, during which the water was replaced on three separate occasions. During the soaking process, the elimination of substandard seeds can be achieved through screening, and the removal of pathogenic microorganisms from the seed surface can be facilitated through rinsing. Furthermore, the seeds demonstrated a substantial reduction in germination time following rapid water absorption. Following the soaking process, the seeds were filtered through gauze and positioned in a horizontal position on the experimental bench to facilitate natural desiccation.

##### 2.1.1.2 Cultivation

The planting site was meticulously selected within a planting box (L × W × H: 1.2 × 0.4 × 0.5 m) situated in the Garden of Medicinal Plants at the Shandong First Medical University (116°8’ 6’’ E, 36°6’ 7’’ N). The soil utilized in the planting box was obtained from the original planting site and possessed a height that was two-thirds of the total height of the box. The planting site is located in the North China Plain, characterized by a warm temperate semi-humid monsoon climate. The soil in this region, developed from alluvial deposits of the Yellow River, is typically calcareous and alkaline due to the relatively low precipitation compared to evaporation, which limits the leaching of calcium and magnesium ions. In order to enhance seed germination conditions, a suitable quantity of dry sheep manure was introduced to the planting substrate before sowing, subsequently amalgamated with the soil. The experimental design involved the implementation of furrow sowing, with the distance between furrows set at 10 centimeters, the depth of each furrow measuring approximately 1 centimeter, and the seeds, which were of the thorn variety, dispersed evenly within the furrows and subsequently covered with a layer of topsoil, which was then moderately compacted. The substrate was subsequently irrigated with the appropriate amount of water until the soil’s surface was moist, after which irrigation was applied irregularly until seed emergence. The aforementioned operation was repeated six times in six planting boxes. Subsequent to emergence, the seedling setting operation was executed to ensure an equal number of NcL in each planting box. Irrigation was implemented on a biweekly basis following the emergence of the seedlings, with a volume of 5 liters of water per application. The frequency of irrigation was augmented in response to hot or arid weather conditions. During the planting phase, Stanley compound fertilizer was utilized as the basal fertilizer to promote the growth of NcL. Periodic weeding treatments were also implemented. The foliage was treated with a 500-fold solution of zinc diclofenac twice during the growing process to prevent and control pests and diseases. To ensure the efficacy of the planting process, regular observation and record-keeping of the NcL’s growth status were implemented. The identification of the herbs was conducted by Associate Professor Xianhui Jia of the School of Pharmacy (Institute of Pharmaceutical Research) at Shandong First Medical University. Professor Jia ascertained that the herbs in question were NcL, a perennial herb in the genus Nepeta, family Labiatae.

#### 2.1.2 Herbal stress treatments

The abiotic stress treatments commenced at the early vegetative stage of NcL. At the onset of the treatments, the plants were uniform in age and size, with an average height of approximately 10 cm and 4–5 pairs of true leaves. All plants belonged to the same sowing batch and were selected for uniformity in age, growth vigor, and size to ensure consistent baseline conditions prior to stress application.

Each treatment group was cultivated as a large cohort of approximately fifty plants to ensure a robust and representative population. From each of these cohorts, a minimum of six individual plants were randomly selected to serve as the biological replicates (n≥6 per treatment) for all subsequent measurements. This random sampling strategy from a large population enhances the generalizability of our findings.

The same control group was used for each stress treatment. Throughout the subsequent experimental period, sampling was consistently performed on plants with comparable growth vigor and size.

##### 2.1.2.1 NaCl stress

Three distinct treatment groups were established with varying salt concentration gradients: 0 (control), 60, and 120 mmol/L. Subsequent to a 45-day period of seedling emergence, the aforementioned random sampling was performed, assigning a minimum of six biological replicates to each treatment group. Changes in soil salt concentrations have been demonstrated to induce an increase in intracellular osmotic pressures in plant cells. This, in turn, results in an accumulation of sodium ions and interferes with the normal metabolic processes and physiological homeostasis of plants(S. Zhao et al., 2021). The present study employed the method of gradually increasing salt concentration to simulate the process of plant adaptation to osmotic stress. The stress concentration gradient was set to 30, 60 mmol/L for the 60 mmol/L group, and to 30, 60, 90, 120 mmol/L for the 120 mmol/L group. Upon reaching the predetermined stress concentration, the stress treatment was administered once a week for a period of 28 days. Subsequent to the attainment of the predefined stress concentration, the stress treatment was administered once weekly for a period of 28 days.

##### 2.1.2.2 K_2_Cr_2_O_7_ stress

Three distinct treatment groups were established with different heavy metal concentration gradients: 0 (control), 15, and 45 mg/L. Subsequent to a 45-day period of seedling emergence, the aforementioned random sampling was performed, assigning a minimum of six biological replicates to each treatment group. In order to circumvent the osmotic stress effect of NcL and to emulate the process of plant acclimatization to osmotic stress, this study employed the method of gradually increasing heavy metal concentration. For the 15 mg/L group, given the low stress concentration, the stress could be given directly; for the 45 mg/L group, the stress concentration gradient was set to 15, 30, 45 mg/L. Upon reaching the predetermined stress concentration, the stress treatment was administered once a week for a period of 28 days.

### 2.2 Biophoton detection

#### 2.2.1 Biophoton detection system

**Figure 1:**
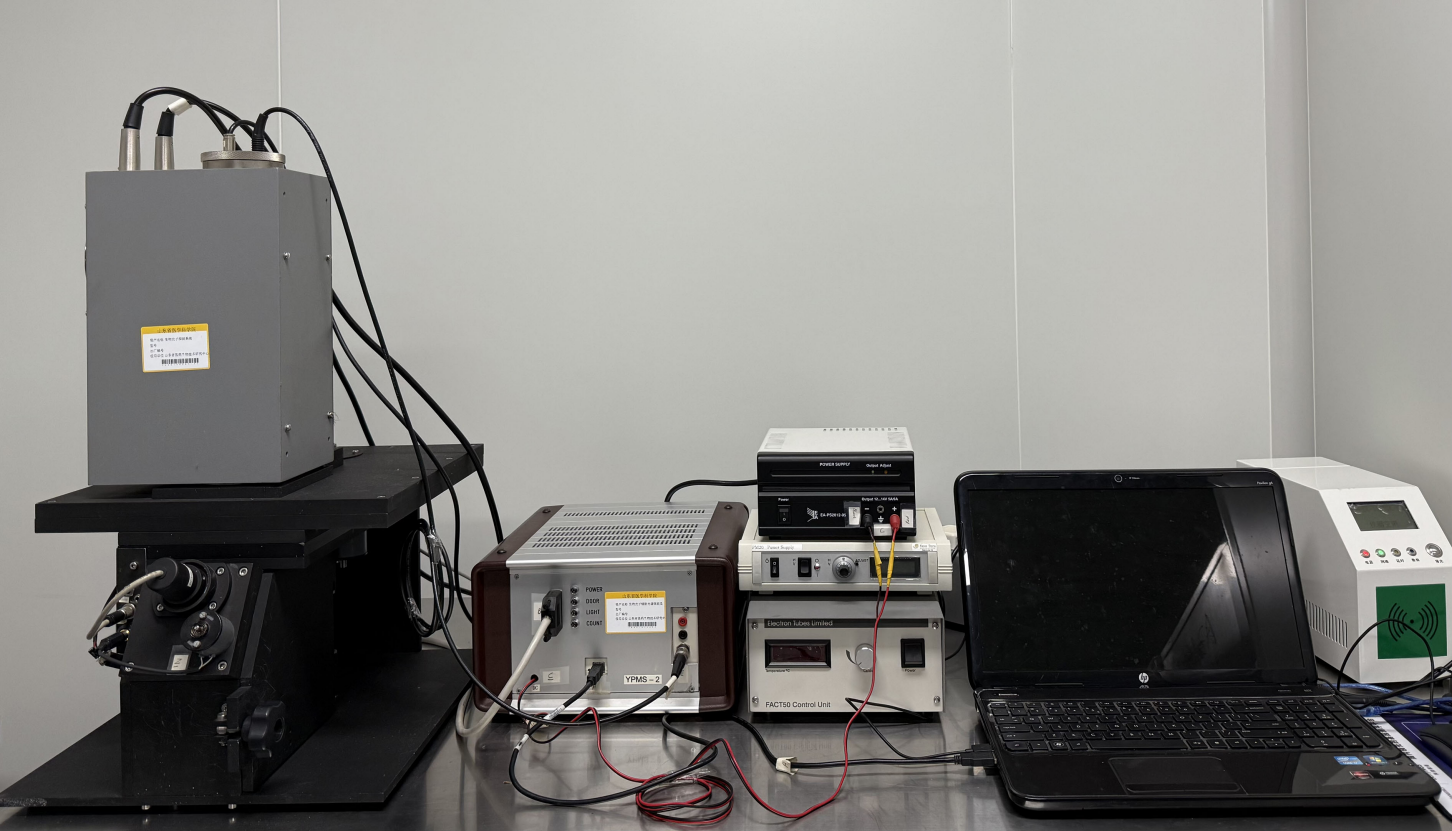
The YPMS-2 biophoton measuring instrument.

The YPMS-2 Biophoton Measuring Instrument, which was built independently (with the help of Meluna, Netherlands), was used as the primary testing equipment in this study. The instrument has been designed based on the synchronized single-photon counting method, which has been demonstrated to offer both high sensitivity and high resolution. The YPMS-2 Biophoton Measuring Instrument consists of several core components, including a computer, a photon counter, a high-voltage power supply, refrigeration equipment, and a dark room, as illustrated in **Figure 1**. The fan system of photomultiplier tube, LED power supply, and refrigeration equipment is integrated above the darkroom, while the sample inlet and sample stage are set below. The sample inlet is fixed by rotary screws to prevent the external light source from entering the darkroom, thus avoiding interference with the test results. The device’s design is meticulous, ensuring the accuracy and reliability of detection.

#### 2.2.2 Sample preparation

In this study, fresh leaves of NcL under varying stress conditions and growth stages were selected as experimental subjects. These subjects were obtained from the planting boxes in the Garden of Medicinal Plants of Shandong First Medical University. In accordance with the stress cycle, the growth process of NcL was divided into four stages. To ensure statistical reliability, data were obtained from six independent biological replicates per treatment. With each measurement involving the random collection of plant tissue from six different plants within a treatment population of approximately fifty individuals. Each sample was then measured three times in parallel as technical replicates to ensure measurement precision. Samples were collected at 7:00-9:00 am and 4:00-6:00 pm to avoid the influence of strong light and extreme weather. The random sampling method was adopted to mitigate the influence of human bias and enhance the representativeness of the samples. Prior to the collection of samples, it is necessary to prepare a foam box that will contain approximately two-thirds of the total volume of ice. It is imperative that the samples be transferred to the ice box for storage immediately after collection and subsequently transferred to the laboratory at the utmost possible speed. Fresh samples were meticulously washed with double-distilled water, and blotting paper was employed to absorb the surface moisture before being placed in the darkroom of the biophoton measuring instrument. The samples were then dark-adapted to the treatment for a duration of one hour, ensuring that they were accurately positioned in the center of the sample stage. During the experiment, it is essential to ensure that the time interval between sample picking and dark adaptation is consistent to maintain the stability of the experimental conditions.

#### 2.2.3 Experimental conditions and procedures

To guarantee that the biophoton measuring instrument operated within its optimal temperature range, the air conditioning unit was set to the cooling mode, thereby maintaining a constant temperature of 20°C prior to the commencement of the experiment. Subsequently, the refrigeration apparatus of the biophoton measuring instrument was activated, and the experiment was initiated when its temperature was reduced to -20°C. The complete cooling process required approximately two hours to complete.

To ensure the accuracy of the biophotonic measurements, a rigorous calibration and dark-count correction protocol was implemented. Prior to each measurement session, the instrument’s background noise was measured for 10 minutes without a sample to establish a stable baseline. The average photon count per second from this measurement was defined as the dark count rate, manually recorded, and subsequently applied as an offset correction in our data analysis script. This value was subtracted from all sample measurements to correct for instrumental noise and ensure that the reported signals originated solely from the biological sample.

With the calibration complete, the formal experiment on plant samples commenced. The measurement interval of the biophoton measuring instrument was set to 1 second for all subsequent data acquisition.

The biophotonic parameters CPS, I_0_, and T were selected for their distinct and complementary abilities to characterize the physiological state of plants under stress. CPS for SPE provides a direct measure of the organism’s real-time metabolic and oxidative activity. As a steady-state parameter, it reflects the continuous production of photons from ongoing biochemical reactions, particularly those involving reactive ROS. I_0_ and T for DL collectively describe the system’s energy storage and dissipation capacity following light excitation. I_0_ represents the initial flux of photons emitted immediately after illumination, which is proportional to the number of excited states populated in the sample. T, the time for the luminescence to decay to a fraction of I_0_, reflects the functional integrity and coherence of the photosynthetic apparatus and other energy-transducing systems; a longer T typically indicates a more ordered and efficient system. The combination of SPE (CPS) and DL (I_0_, T) parameters thus offers a comprehensive profile, capturing both the dynamic oxidative metabolism and the photophysical properties related to the plant’s energy budget, which are anticipated to be perturbed by abiotic stress.

The detailed calculation formulas for CPS, I_0_, and T, along with the derivation of the Gu parametric model, are provided in the **Supplementary Materials** (Section S1). In brief, CPS was calculated by subtracting the dark-count rate from the average photon count, while I_0_ and T were derived from the non-linear fitting of the delayed luminescence decay curve. To account for inter-sample variations, the raw CPS and I_0_ values were standardized based on sample weight and thickness.

### 2.3 Determination of physiological and biochemical indexes

Fresh NcL leaves were collected, rinsed with double-distilled water, and blotted dry with absorbent paper.

#### 2.3.1 Determination of the photosynthetic pigments content

Leaf tissue (0.1 g) was minced and extracted with 15 mL of an acetone-ethanol (1:1, v/v) mixture in a 50-mL centrifuge tube. The samples were kept in complete darkness for 24 hours, with periodic mixing every 8 hours. The absorbance of the extracts was measured at 474, 642, and 665 nm using a U-3900H UV-Vis spectrophotometer, with the extraction solvent as a blank. The contents of chlorophyll a, chlorophyll b, total chlorophyll, and carotenoids were calculated according to established formulas, the detailed calculation formulas for photosynthetic pigments are provided in the **Supplementary Materials** (Section S2).

#### 2.3.2 Determination of the relative electrical conductivity

The samples were collected on five separate occasions at various locations on the treated leaves using a hole punch (d = 6 mm). The collected samples were subsequently placed in 100-mL conical flasks containing 30 mL of double-distilled water. The samples were then continuously extracted in a constant temperature shaker (150 rpm) for a period of 12 hours. The specific determination steps and calculation formulas are provided in the **Supplementary Materials** (Section S3).

### 2.4 Determination of oxidative stress indexes

#### 2.4.1 Determination of the rate of ROS production

Leaf tissue (0.1 g) was homogenized in 1 mL of ice-cold Tris-HCl buffer containing phenylmethylsulfonyl fluoride. The homogenate was centrifuged at 600 × g for 5 min at 4°C. The supernatant was then centrifuged at 11,000 × g for 10 min at 4°C. The pellet was resuspended in 200 μL of Tris-HCl buffer for analysis. According to **Table 1**, mitochondrial suspensions and reagents were incubated in a black 96-well plate at 37°C for 15 minutes. Fluorescence intensity (excitation 499 nm, emission 521 nm) was recorded for 10 minutes using a SpectraMax iD5 ELISA reader. The specific determination steps and calculation formulas are provided in the **Supplementary Materials** (Section S4).

**Table 1:** Composition of reagents in blank and measurement tubes.

Reagent (μL)	Blank tube	Measuring tube
Mitochondrial suspension	0	20
Tris-HCl buffer solution	20	0
Malic acid	50	50
Pyruvate	50	50
Succinic acid	50	50
Fluorescent probe - reduced dichlorofluorescein	30	30

#### 2.4.2 Determination the content of the malondialdehyde

Leaf tissue (0.1 g) was homogenized in 1 mL of MDA extraction buffer on ice. The homogenate was centrifuged at 8,000 × g for 10 min at 4°C. The supernatant was collected for analysis. According to **Table 2**, samples were mixed with MDA detection working solution, heated at 100°C for 60 minutes, and cooled. After centrifugation at 10,000 × g for 10 min, the supernatant was collected. Absorbance was measured at 532 nm and 600 nm. The specific determination steps and calculation formulas are provided in the **Supplementary Materials** (Section S5).

**Table 2:** Composition of reagents in blank and measurement tubes.

Reagent (μL)	Blank tube	Measuring tube
MDA detection working fluid	300	300
Distilled water	100	0
Sample	0	100

#### 2.4.3 Determination the content of the superoxide anion (O_2_•-)

Leaf tissue (0.1 g) was homogenized in O_2_•- extraction buffer on ice. The homogenate was centrifuged at 12,000 × g for 10 min at 4°C. The supernatant was collected for analysis. Standard solutions were prepared as described. According to **Table 3**, samples, standards, and reagents were incubated at 37°C for 20 minutes. After adding Reagent 2 and Reagent 3 and incubating again, chloroform was added and the mixture was centrifuged. The absorbance of the aqueous phase was measured at 530 nm. The specific determination steps and calculation formulas are provided in the **Supplementary Materials** (Section S6).

**Table 3:** Composition of reagents in blank, measurement and standard tubes.

Reagent (μL)	Blank tube	Measuring tube	Standard tube
Reference material	0	0	100
Sample	0	100	0
Distilled water	100	0	0

With caution, 200 microliters of the upper aqueous phase should be transferred to a 96-well plate, and the absorbances of each group should be measured at a wavelength of 530 nanometers. The absorbances should be documented as A_b_, A_m_, and A_s_ respectively. The O_2_•- content (μmol/g) is calculated according to formula 18, where W is the sample mass. The calculation formula is as follows:

### 2.5 Content determination of PG based on UPLC

#### 2.5.1 Sample preparation for content determination

Mature NcL samples were collected, washed, air-dried, and ground into powder. The powder was sieved (24 mesh) and stored in sealed bags. Powder (0.5 g) was extracted with 10 mL methanol by ultrasonication (250 W, 50 kHz) for 20 minutes. After filtration, the residue was re-extracted with 10 mL methanol. The combined filtrates were diluted to 25 mL with methanol in a volumetric flask to obtain the test solution.

#### 2.5.2 UPLC analysis

The UPLC analysis was conducted on a Waters ultra performance liquid chromatograph (Waters, Milford, MA, USA) equipped with an ACQUITY UPLC BEH C18 column (2.1 mm × 150 mm, 1.7 μm) as the stationary phase, the mobile phase consisted of a methanol-water mixture (83:17), the detection wavelength was set at 252 nm, and the detection time was 30 minutes, the flow rate was 0.2 milliliters per minute, the injection volume was 7.5 microliters, the column temperature was 29.5 degrees Celsius, and the cuvette temperature was 25 degrees Celsius. The method demonstrated commendable precision, stability, and reproducibility for PG, and the linear regression formula for PG was successfully obtained.

### 2.6 Data analysis

A one-way analysis of variance (ANOVA) was conducted using GraphPad Prism 8.0 software on experimental data collected during the mature stage (i.e., the final growth stage) to analyze the significance of differences between data sets. The underlying assumptions of ANOVA, including homogeneity of variances (verified via the Brown-Forsythe test) and approximate normality of residuals, were confirmed to be met by the dataset, justifying the use of this parametric test. The Pearson correlation coefficient analysis was performed using Origin 2021 software to examine the correlation characteristics between data sets and to visualize the results. All statistical analyses were conducted with a significance level set at α = 0.05. When the p-value was ≤ α, the results were considered to have statistically significant differences.

## 3 Result

### 3.1 Results of biophoton characteristics

#### 3.1.1 Biophoton Characteristics of NcL under different stress conditions

##### 3.1.1.1 SPE characteristics

**Figure 2:**
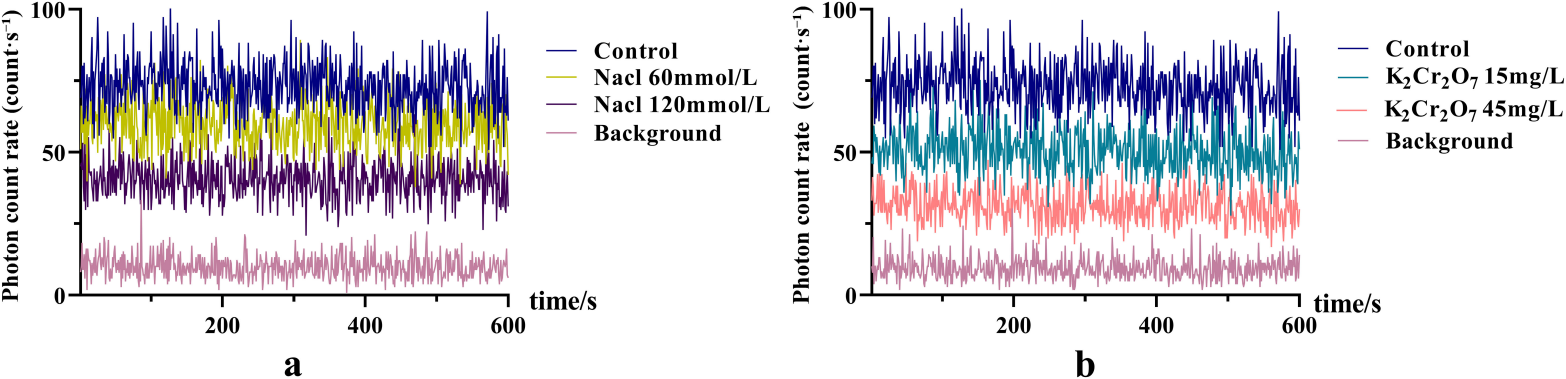
The SPE characteristics of *Nepeta cataria L.* under different stress conditions.

The SPE characteristics of NcL under different stress conditions are shown in **Figure 2**. The photon intensity under steady-state conditions was lower in the stress-treated groups compared to the control group.

##### 3.1.1.2 DL characteristics

**Figure 3:**
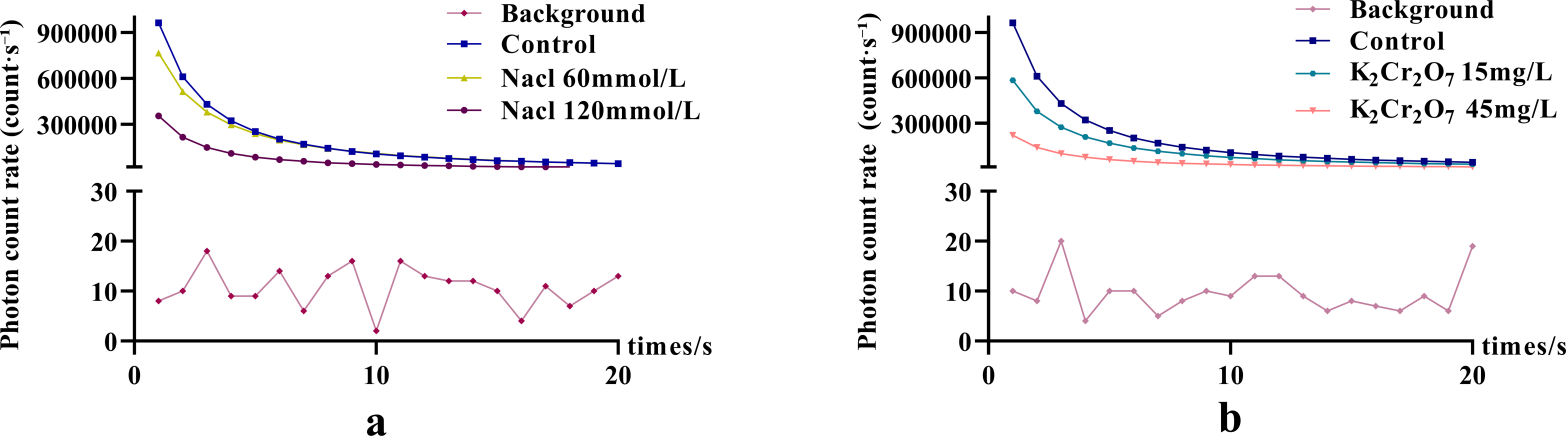
The DL characteristics of *Nepeta cataria* L. under different stress conditions.

The DL decay curves of NcL under varying stress conditions are shown in **Figure 3**. The I_0_ in all stress groups was significantly lower than that in the control group.

#### 3.1.2 Analysis of bioluminescence parameters of NcL under different concentrations of salt stress

**Figure 4:**
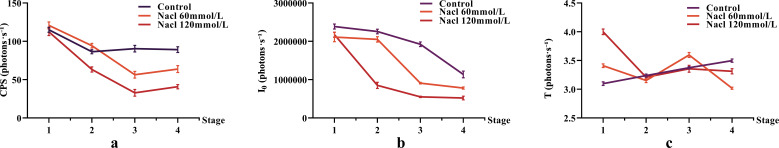
The changes in bioluminescence parameters of *Nepeta cataria* L. under different salt concentrations stress with treatment time, the mean and standard deviation(SD) values for each group are provided in **Supplementary Table 1**.

The bioluminescence parameters (CPS, I_0_, T) of NcL under different salt stress conditions are shown in **Figure 4**. The CPS values in the salt-stressed groups were lower than those in the control group in the final two stages. The I_0_ values in the salt-stressed groups were lower than those in the control group throughout the process. The T parameter did not show a clear trend.

**Figure 5:**
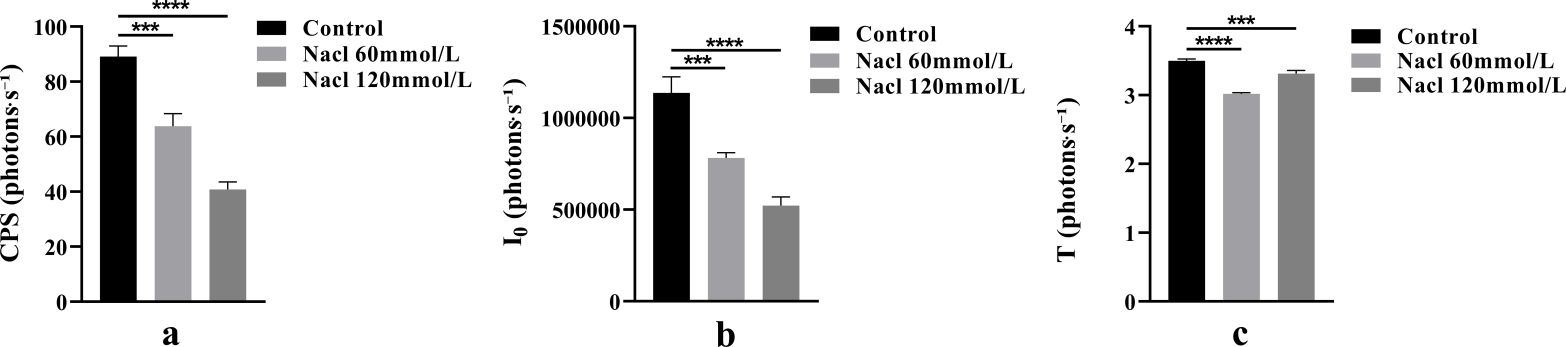
The One-way ANOVA analysis results of bioluminescence parameters in fresh samples of *Nepeta cataria* L. under different salt concentrations stress (***P ≤ 0.001; ****P ≤ 0.0001), the mean and SD values for each group are provided in **Supplementary Table 2**.

One-way ANOVA results of bioluminescence parameters are shown in **Figure 5**. The CPS, I_0_, and T values were as follows: control group (89.16, 1.14 × 10^6^, 3.50), 60 mmol/L salt stress group (63.80, 7.82 × 10^5^, 3.02), and 120 mmol/L salt stress group (40.77, 5.22 × 10^5^, 3.31).

#### 3.1.3 Analysis of bioluminescence parameters of NcL under different concentrations of heavy metal stress

**Figure 6:**
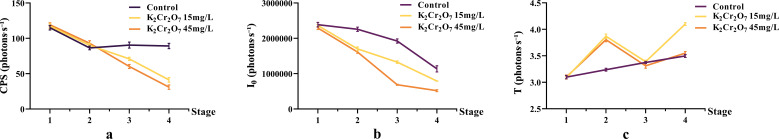
The changes in bioluminescence parameters of *Nepeta cataria* L. under different heavy metal concentrations stress with treatment time, the mean and SD values for each group are provided in **Supplementary Table 3**.

The bioluminescence parameters (CPS, I_0_, T) of NcL under different heavy metal stress conditions are shown in **Figure 6**. The CPS values in the heavy metal-stressed groups were lower than those in the control group in the later stages. The I_0_ values in the heavy metal-stressed groups were lower than those in the control group throughout the process. The T parameter did not show a clear trend.

**Figure 7:**
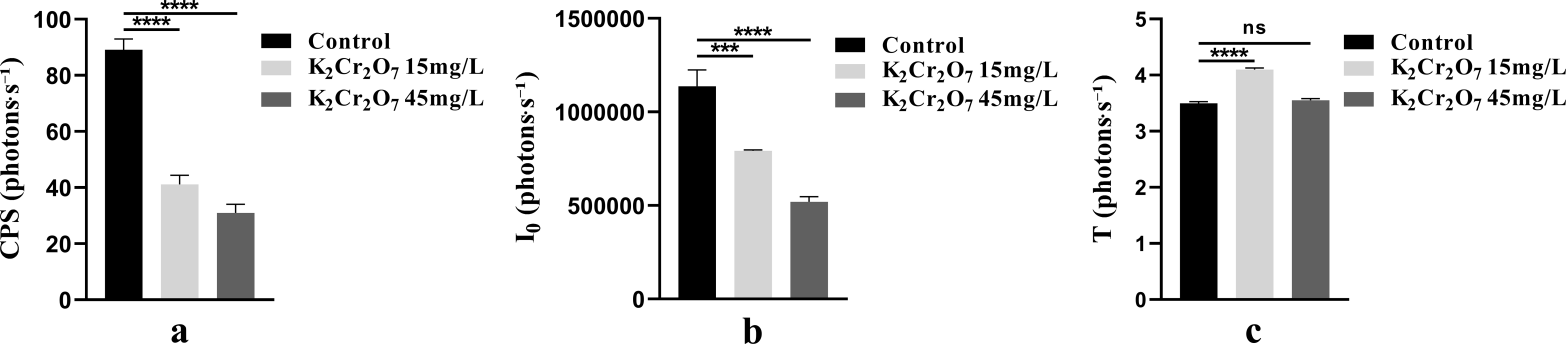
The One-way ANOVA analysis results of bioluminescence parameters in fresh samples of *Nepeta cataria* L. under different heavy metal concentrations stress (ns>0.05; ***P ≤ 0.001; ****P ≤ 0.0001), the mean and SD values for each group are provided in **Supplementary Table 4**.

One-way ANOVA results of bioluminescence parameters under heavy metal stress are shown in **Figure 7**. The CPS, I_0_, and T values were as follows: control group (89.16, 1.14 × 10^6^, 3.50), 15 mg/L heavy metal stress group (41.15, 7.92 × 10^5^, 4.10), and 45 mg/L heavy metal stress group (30.96, 5.19 × 10^5^, 3.55).

### 3.2 Results of physiological and biochemical indexes

#### 3.2.1 Photosynthetic pigments content

**Figure 8:**
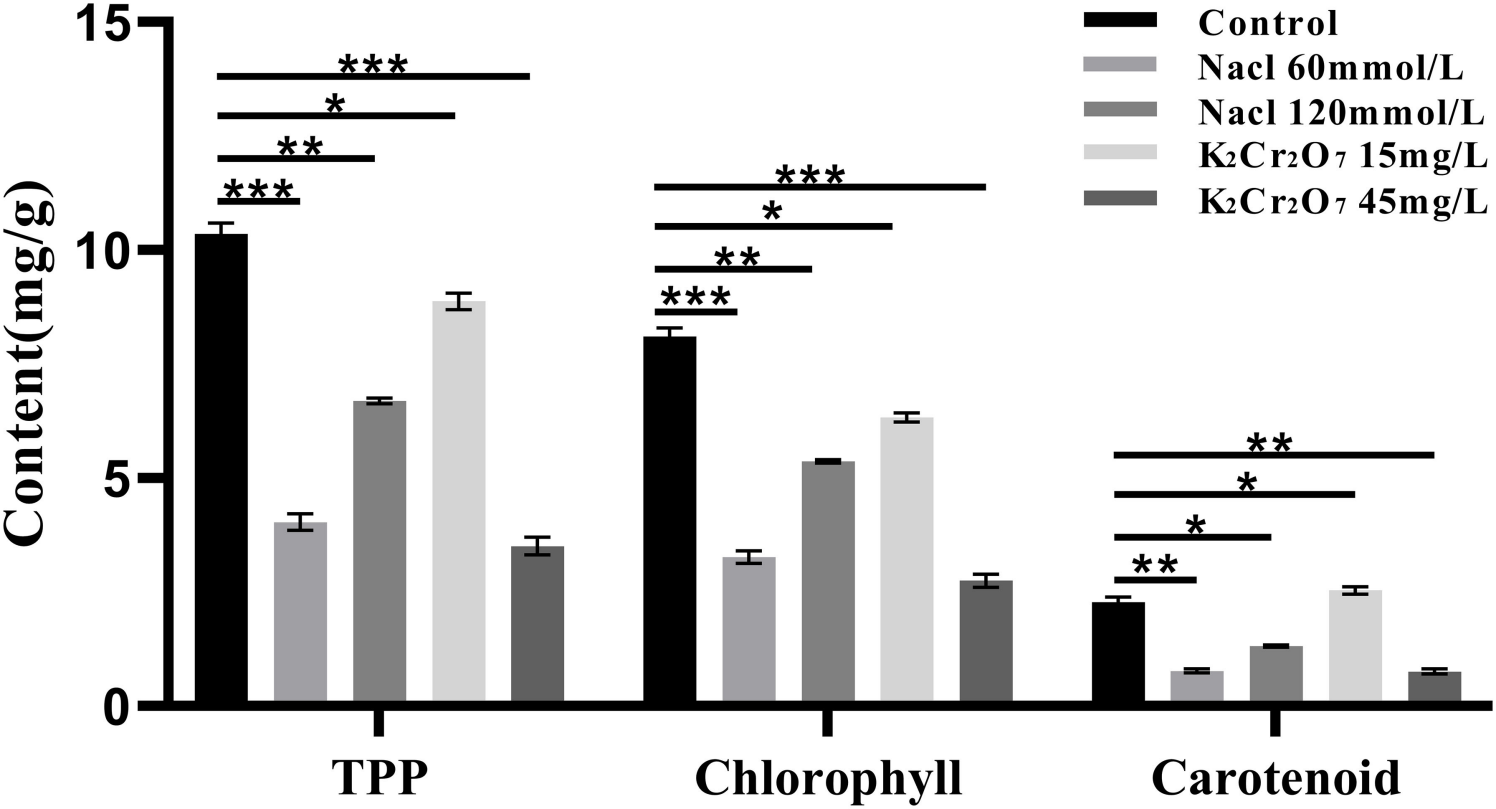
The One-way ANOVA analysis results of photosynthetic pigments content in fresh samples of *Nepeta cataria* L. under different stresses (*P ≤ 0.05; **P ≤ 0.01; ****P ≤ 0.0001), the mean and SD values for each group are provided in **Supplementary Table 5**.

The contents of total photosynthetic pigments (TPP), chlorophyll, and carotenoids in fresh NcL samples are presented in **Figure 8**. TPP content: control group (10.35 mg/g); salt stress groups: 60 mmol/L (6.69 mg/g), 120 mmol/L (4.04 mg/g); heavy metal stress groups: 15 mg/L (8.88 mg/g), 45 mg/L (3.51 mg/g);Chlorophyll content: control group (8.11 mg/g); salt stress groups: 60 mmol/L (5.37 mg/g), 120 mmol/L (3.27 mg/g); heavy metal stress groups: 15 mg/L (6.34 mg/g), 45 mg/L (2.75 mg/g); Carotenoid content: control group (2.28 mg/g); salt stress groups: 60 mmol/L (1.33 mg/g), 120 mmol/L (0.76 mg/g); heavy metal stress groups: 15 mg/L (2.54 mg/g), 45 mg/L (0.77 mg/g).

#### 3.2.2 REC

**Figure 9:**
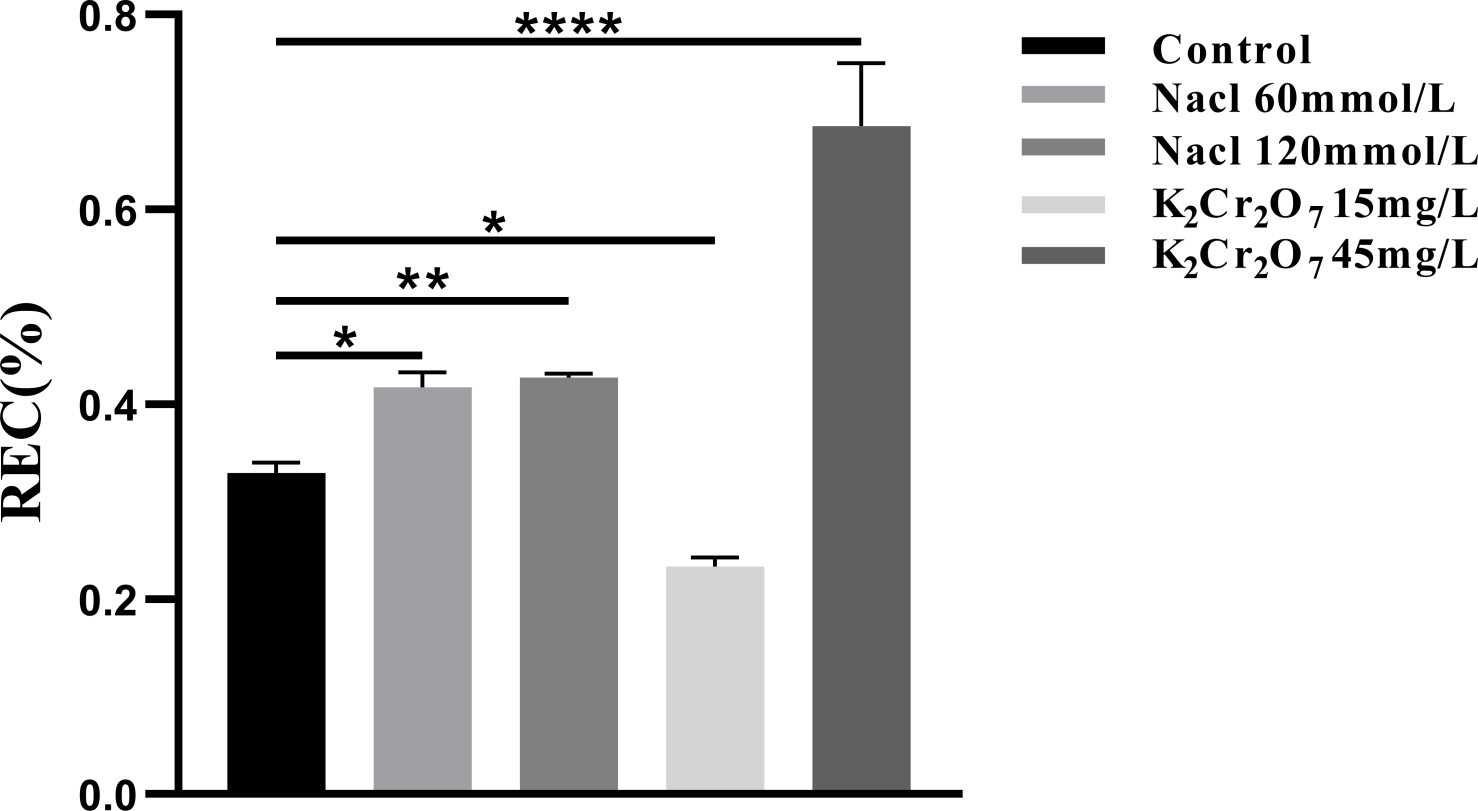
The One-way ANOVA analysis results of REC in fresh samples of *Nepeta cataria* L. under different stresses (*P ≤ 0.05; **P ≤ 0.01; ****P ≤ 0.0001), the mean and SD values for each group are provided in **Supplementary Table 6**.

The REC of each experimental group is presented in **Figure 9**. The REC values were as follows: control group (0.33%), 60 mmol/L salt stress group (0.42%), 120 mmol/L salt stress group (0.43%), 15 mg/L heavy metal stress group (0.23%), and 45 mg/L heavy metal stress group (0.69%).

### 3.3 Results of oxidative stress indexes

#### 3.3.1 The rate of ROS production

**Figure 10:**
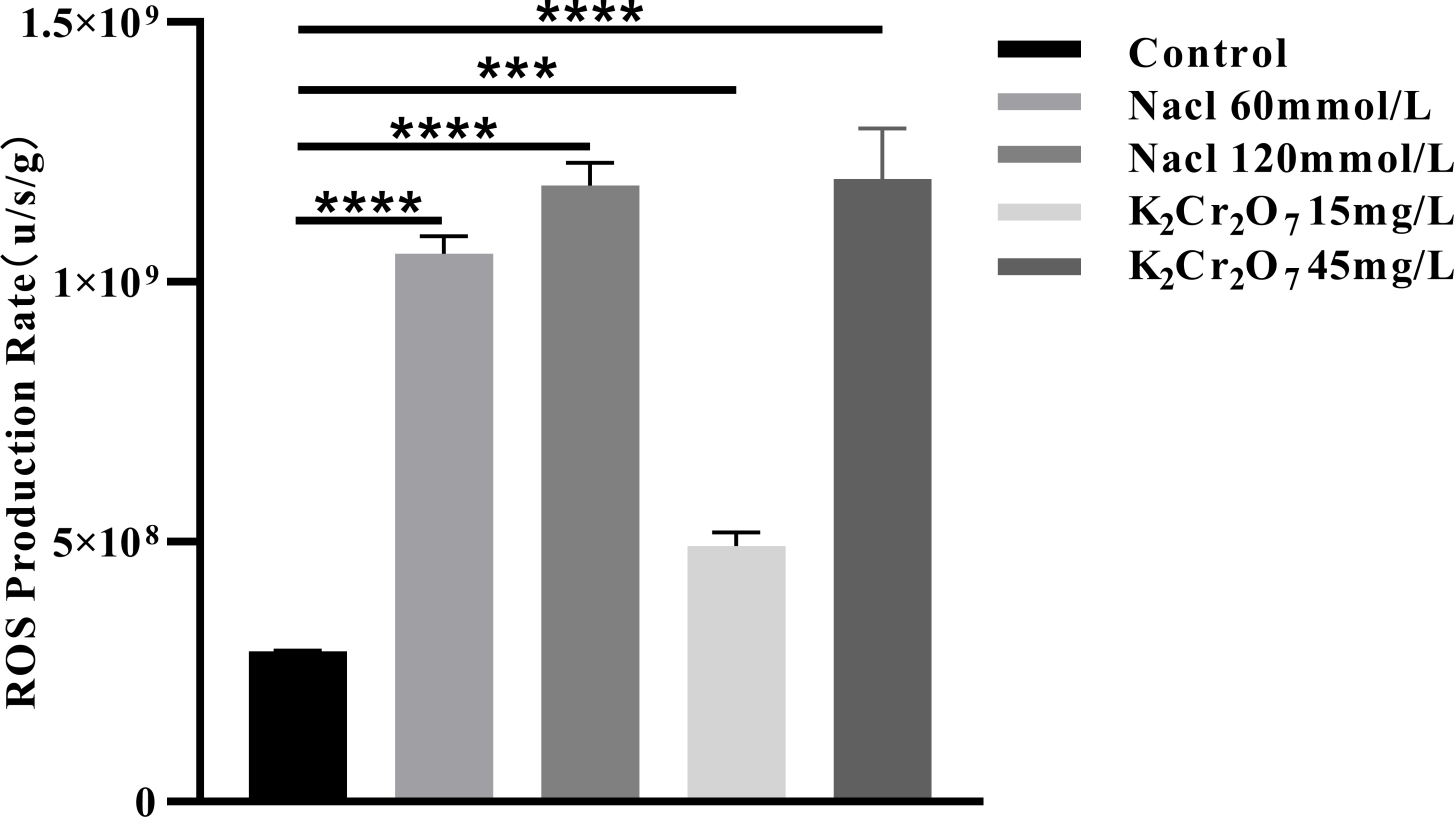
The One-way ANOVA analysis results of the ROS production rate in fresh samples of *Nepeta cataria* L. under different stresses (***P ≤ 0.001; ****P ≤ 0.0001), the mean and SD values for each group are provided in **Supplementary Table 7**.

The rates of ROS production in fresh NcL samples are presented in **Figure 10**. The values were as follows: control group (2.88 × 10^8^ u/s/g), 60 mmol/L salt stress group (1.05 × 10^9^ u/s/g), 120 mmol/L salt stress group (1.19 × 10^9^ u/s/g), 15 mg/L heavy metal stress group (4.92 × 10^8^ u/s/g), and 45 mg/L heavy metal stress group (1.19 × 10^9^ u/s/g).

#### 3.3.2 MDA content

**Figure 11:**
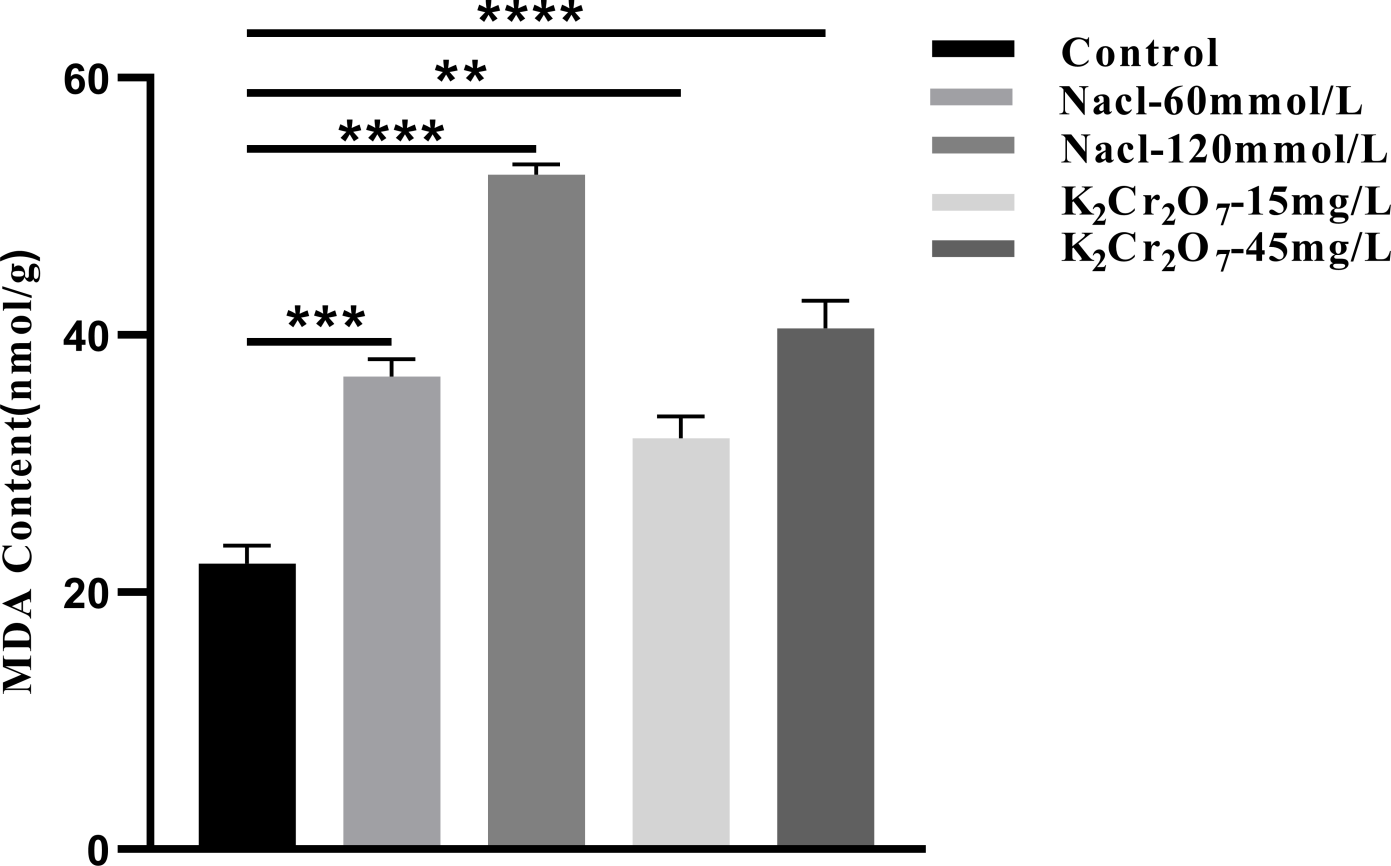
The One-way ANOVA analysis results of the MDA content in fresh samples of *Nepeta cataria* L. under different stresses (**P ≤ 0.01; ***P ≤ 0.001; ****P ≤ 0.0001), the mean and SD values for each group are provided in **Supplementary Table 8**.

The MDA content in fresh NcL samples is presented in **Figure 11**. The values were as follows: control group (22.20 nmol/g), 60 mmol/L salt stress group (36.79 nmol/g), 120 mmol/L salt stress group (52.49 nmol/g), 15 mg/L heavy metal stress group (31.95 nmol/g), and 45 mg/L heavy metal stress group (40.50 nmol/g).

#### 3.3.3 O_2_•- content

**Figure 12:**
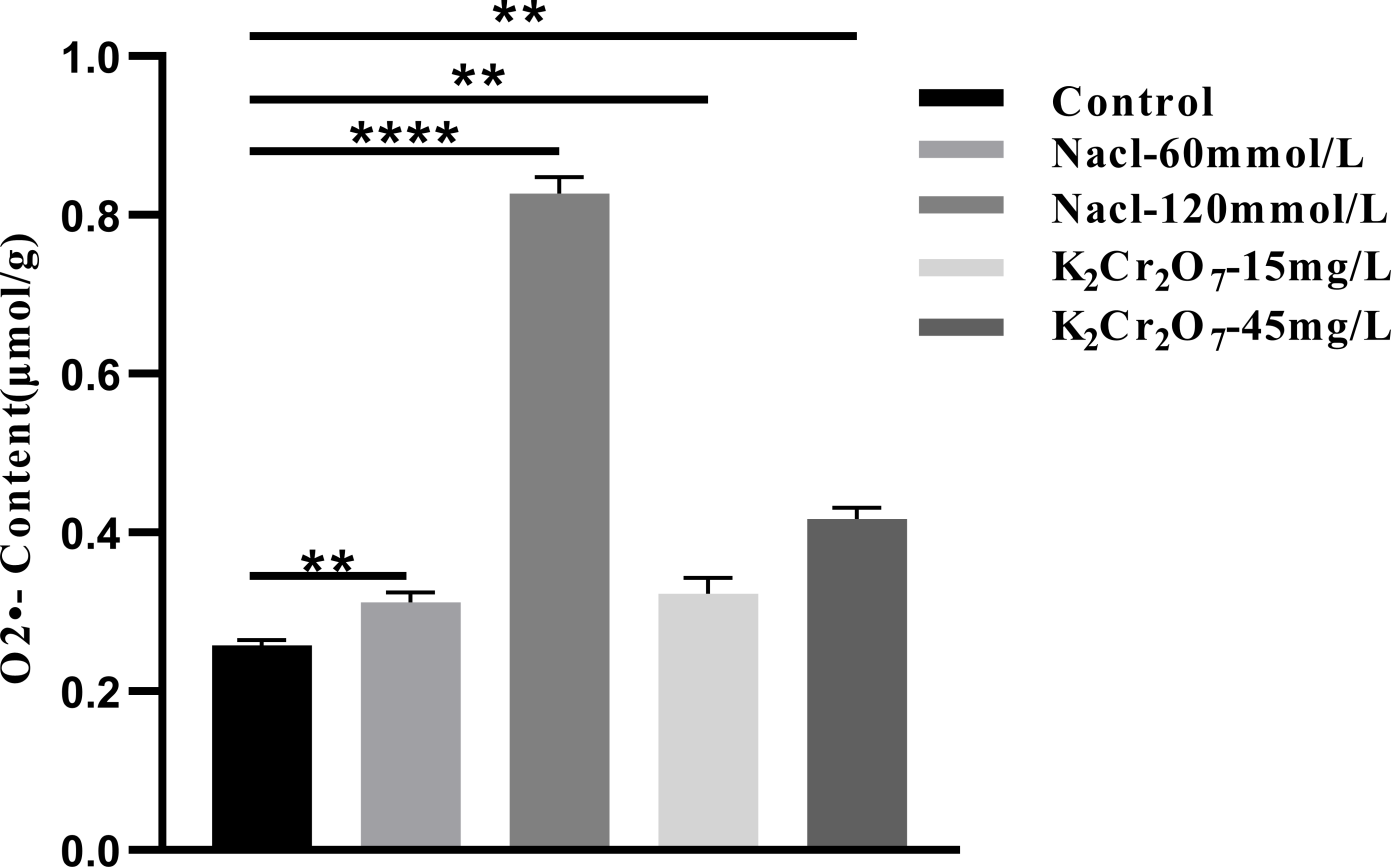
The One-way ANOVA analysis results of the O_2_•- content in fresh samples of *Nepeta cataria* L. under different stresses (**P ≤ 0.01; ****P ≤ 0.0001), the mean and SD values for each group are provided in **Supplementary Table 9**.

The O_2_•- content in fresh NcL samples is presented in **Figure 12**. The values were as follows: control group (0.26μmol/g), 60 mmol/L salt stress group (0.37 μmol/g), 120 mmol/L salt stress group (0.86 μmol/g), 15 mg/L heavy metal stress group (0.32 μmol/g), and 45 mg/L heavy metal stress group (0.43 μmol/g).

### 3.4 Results of PG content test

#### 3.4.1 Method validation for PG quantification

The HPLC analysis for PG quantification was rigorously validated. The target compound, PG, was well-separated with a retention time of 3.2 minutes. The calibration curve, constructed over a concentration range of 0.01 to 1 mg/g, exhibited excellent linearity, represented by the equation y=14163567.69x–121611.55 with a coefficient of determination (R^2^) of 0.9991. The limit of detection and limit of quantification were derived from the calibration curve using the recognized method based on the standard deviation of the response and the slope, and were found to be 0.003 mg/g and 0.01 mg/g, respectively.

#### 3.4.2 PG content

**Figure 13:**
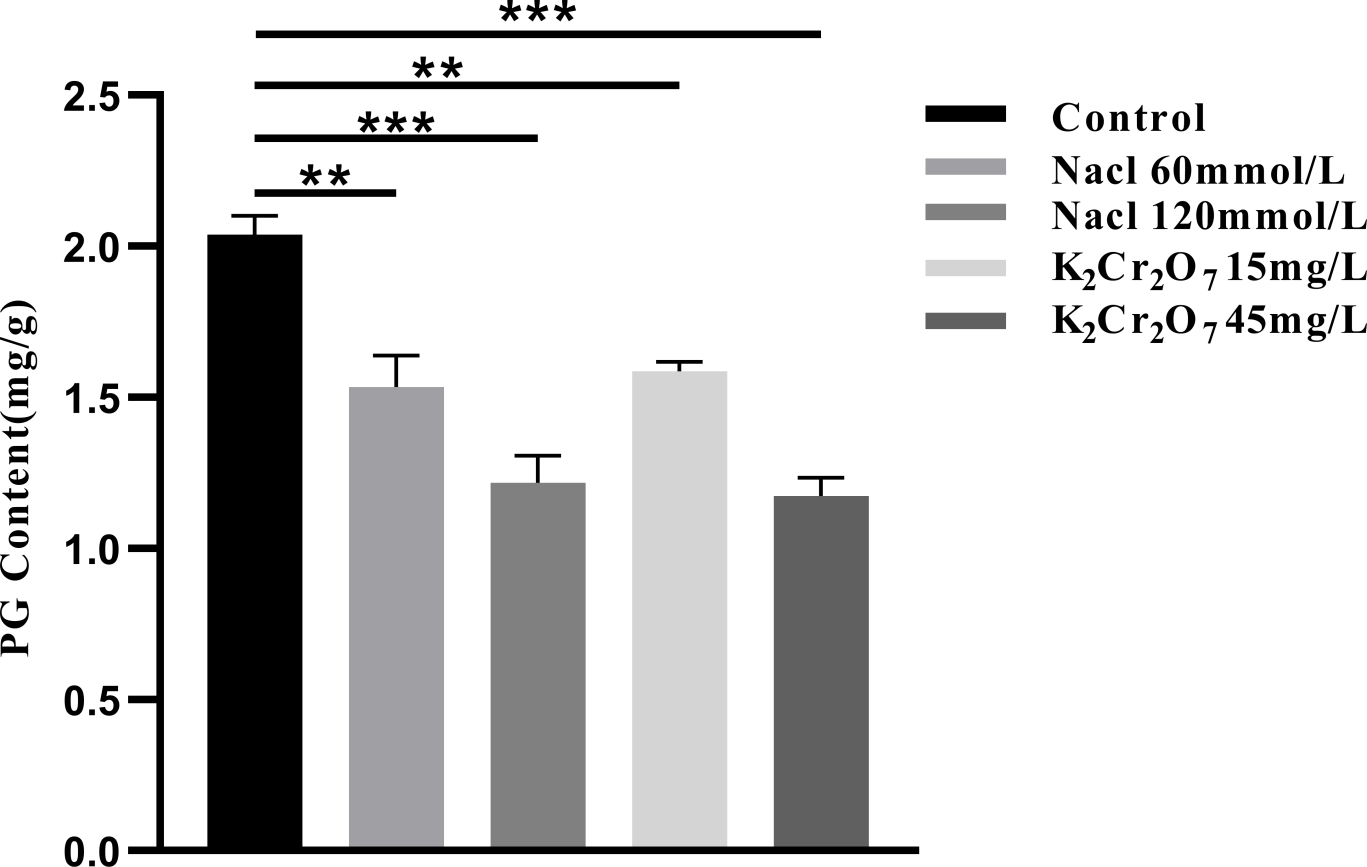
The One-way ANOVA analysis results of the pulegone content in fresh samples of Ne*peta cataria* L. under different stresses (**P ≤ 0.01; ***P ≤ 0.001), the mean and SD values for each group are provided in **Supplementary Table 10**.

The PG content in NcL samples is presented in **Figure 13**. The values were as follows: control group (2.04 mg/g), 60 mmol/L salt stress group (1.53 mg/g), 120 mmol/L salt stress group (1.22 mg/g), 15 mg/L heavy metal stress group (1.59 mg/g), and 45 mg/L heavy metal stress group (1.18 mg/g).

### 3.5 Correlation analysis based on the above results

**Figure 14:**
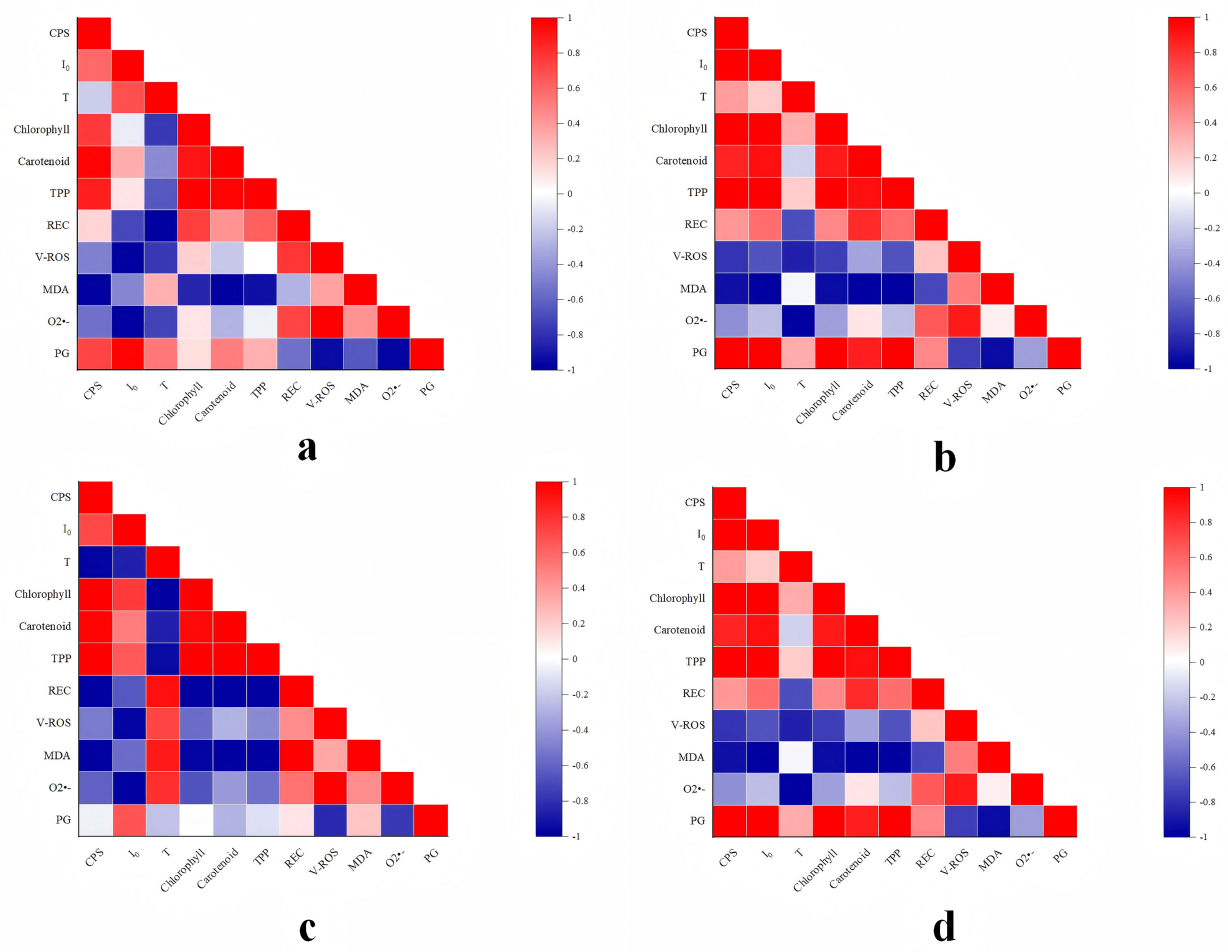
Correlation analysis of experimental results of *Nepeta cataria* L. samples under different stress conditions.

In light of the experimental findings outlined above, a correlation analysis method was employed, utilizing Pearson’s correlation coefficient to examine the relationships among the bioluminescence characteristics, physiological and biochemical indexes, oxidative stress indexes, and pulegone content in NcL samples under diverse stress conditions. The findings were then visualized (**Figure 14**) as follows:

In conditions of salt stress below 60 mmol/L (**Figure 14a**), a significant positive correlation was observed between CPS and photosynthetic pigment content. No significant correlation was found between CPS and REC, however negative correlations were identified between CPS and all oxidative stress indexes. A significant positive correlation was also identified between CPS and PG content. I_0_ demonstrated a moderate correlation with photosynthetic pigment content, negative correlations with REC and all oxidative stress indexes, and a significant positive correlation with PG content. T showed significant negative correlations with all physiological and biochemical indexes and oxidative stress indexes, while exhibiting a positive correlation with PG. In the context of salt stress levels below 120 mmol/L (**Figure 14b**), a significant positive correlation was observed between CPS and I_0_ on the one hand, and all physiological and biochemical indexes and PG content on the other. Conversely, a significant negative correlation was identified between CPS and I_0_, and all oxidative stress indexes. T exhibited a moderate correlation with photosynthetic pigment content, significant negative correlations with REC, ROS production rate, and O_2_•- content, no correlation with MDA, and a strong correlation with PG content.

In conditions of heavy metal stress below 15 mg/L (**Figure 14c**), a significant positive correlation was observed between CPS and photosynthetic pigment content. A significant negative correlation was observed between CPS and both REC and all oxidative stress indexes. No correlation was observed between CPS and PG content. In contrast, I_0_ exhibited a significant positive correlation with photosynthetic pigment content, a significant negative correlation with REC and all oxidative stress indexes, and a moderate correlation with PG content. T showed a significant negative correlation with chlorophyll content, a significant positive correlation with REC and oxidative stress indexes, and a weak correlation with borneol content. In the context of concentrations below 45 mg/L of heavy metals (**Figure 14d**), a notable positive correlation was observed between CPS and I_0_ on the one hand, and chlorophyll pigment and borneol content on the other. Furthermore, moderate correlations were identified between CPS and I_0_, as well as between CPS and REC. In addition, a significant negative correlation was demonstrated between CPS and I_0_, on the one hand, and all oxidative stress indexes, on the other. T exhibited moderate correlation with chlorophyll pigment content, significant negative correlations with REC, ROS production rate, and O_2_•- content, no significant correlation with MDA content, and moderate correlation with PG content.

## 4 Discussion

Our results demonstrate that both salt and heavy metal stress significantly altered the biophysical and biochemical properties of NcL. The biophoton emission parameters, namely CPS and I_0_, were markedly suppressed under stress. Specifically, under 60 and 120 mmol/L salt stress, CPS decreased by 28.44% and 54.27%, and I_0_ decreased by 31.40% and 54.12%, respectively. A similar trend was observed under heavy metal stress, with CPS and I_0_ decreasing by 53.85% and 65.28% at 15 mg/L, and by 54.27% and 54.47% at 45 mg/L, respectively. These declines in biophoton emission were accompanied by substantial physiological disruption. The contents of photosynthetic pigments showed declines of up to 66%, with TPP, chlorophyll, and carotenoids decreasing by 35%, 61%, 14%, 66%; 34%, 60%, 22%, 66%; and 42%, 67%, -11%, 66% across the stress groups, respectively. Meanwhile, REC increased by 27.3%, 30.3%, -30.3%, and 110.6%. Furthermore, a pronounced oxidative stress response was triggered, as evidenced by the increase in ROS production rate (264.58%, 313.20%, 70.83%, 313.20%), MDA content (65.72%, 136.44%, 44.01%, 82.43%), and O_2_•- accumulation (42.31%, 230.77%, 23.08%, 65.38%). Critically, the content of the key bioactive compound PG was also significantly compromised, showing reductions of 24.78%, 40.19%, 22.18%, and 42.35% across all stress groups. These comprehensive alterations in both biophoton emission and a suite of physiological and biochemical indicators, oxidative indexes and PG content strongly suggest that stress severely disrupted the internal metabolic activity of NcL.

The suppression of both SPE and DL under abiotic stress observed in our study aligns with the findings from prior biophoton research on medicinal plants. For instance, similar decrease in DL intensity and photon count was reported in *Leonurus japonicus* Houtt. under drought stress, and was also interpreted as a consequence of impaired photosynthesis and overall metabolic decline (Cao et al., 2023). However, our study provides a more integrated perspective by simultaneously linking these biophotonic changes to a comprehensive suite of oxidative stress markers and the content of a key bioactive compound (PG), which has been scarcely reported in previous work.

The results of the bioluminescence characteristic detection indicate that, in comparison with the control group, there are significant differences in the bioluminescence characteristics of NcL under different stress conditions, particularly in the representative parameters CPS for SPE and I_0_ and T for DL. At the final stage of the growth process, the control group exhibited significantly higher values for CPS, I_0_, and T than all stress groups, indicating that the bioluminescence intensity of the control group was significantly higher than that of the stressed groups. The metabolic luminescence theory hypothesizes that the SPE characteristics of living organisms and their representative parameter CPS are closely linked to internal redox metabolic activities. In contrast, the DL characteristics and their representative parameter I_0_ are intrinsically linked to the process of biomolecules transitioning from high-energy states to low-energy states. During metabolism, a significant quantity of released energy is converted into biophotons, which are subsequently emitted in the form of bioluminescence (Hideg et al., 1990; Popp, 2003). When exposed to external light sources, biomolecules within living organisms are excited, causing more biomolecules to transition into high-energy states. Consequently, the initial intensity of I_0_ is elevated. Furthermore, living organisms in different states exhibit varying degrees of responsiveness to external stimuli. Consequently, the intensity of metabolic activity within living organisms can be characterized by the level of I_0_ (Gang et al., 2009, 2011). During the late growth stage, salt stress at varying concentrations and heavy metal stress exerted differing degrees of impact on NcL. The CPS, I_0_, and T values in all stress groups were found to be significantly lower than those in the control group. This finding suggests that the growth state of NcL underwent substantial alterations when subjected to stress conditions. Stress conditions significantly suppressed normal metabolic activities within the plant, leading to a substantial reduction in the number of biomolecules in high-energy states. Concurrently, the available energy reserves proved insufficient to sustain normal metabolic functions, consequently resulting in diminished bioluminescence intensity. This finding is consistent with the prevailing scientific consensus.

A comparison of the growth status of NcL samples under different stress conditions at the same growth stage revealed significant differences when compared with the control group. This finding suggests that the growth of the samples was inhibited as a result of the stress conditions to which they were exposed. The size of REC is closely related to cell membrane permeability, directly reflecting membrane permeability and damage levels, and also serves as a crucial indicator for assessing plant stress resistance (Liye et al., 2024; Xiaoxiao et al., 2024). Stress conditions or specific environments that are not conducive to plant growth have been shown to have a significant impact on the internal environment of the plant, particularly with regard to the integrity and permeability of its cell membrane structure and function. In typical circumstances, this process typically results in an increase in cell membrane permeability, which in turn leads to substantial outward ion efflux from plant cells. The results of the REC detection analysis indicate that, with the exception of the 15 mg/L heavy metal stress group, where REC was marginally lower than in the control group, REC in all other stress groups was significantly higher than in the control group. This finding suggests that, with the exception of the 15 mg/L heavy metal stress group, there was a significant increase in cell membrane permeability of NcL samples in various salt stress groups and the 45 mg/L heavy metal stress group. The results of this study indicate that the normal physiological structure and function of NcL leaf cells were disrupted under stress treatment.

The oxidative stress status of NcL samples was found to be significantly affected by salt stress and heavy metal stress at varying concentrations. In comparison with the control group, ROS production rates were significantly elevated in all groups experiencing stress. For instance, a marked increase of approximately 2.0- to 2.5-fold was observed under severe stress conditions (e.g., 120 mmol/L NaCl). It is evident that ROS function as pivotal signaling molecules, thereby facilitating expeditious cellular responses to diverse stimuli. They play a pivotal role in stress perception and the activation of stress response networks (Choudhury et al., 2017; Mittler et al., 2022; Suzuki et al., 2012). Under normal conditions, the equilibrium between ROS production and consumption is crucial for plant growth and development, as well as the accumulation of organic matter (Poljsak et al., 2013). In conditions of stress, there is a marked increase in the rate of ROS production. This phenomenon can be attributed to two factors. Firstly, it represents the plant’s intrinsic active response to stress. Secondly, stress disrupts the dynamic equilibrium between ROS production and consumption, leading to excessive ROS accumulation (Akter et al., 2021). This excessive ROS directly drives lipid peroxidation, a key mechanism of oxidative injury.

Concurrently, the MDA content in NcL samples was found to be considerably influenced by the imposed stress conditions. MDA has been shown to reflect the extent of oxidative damage to cell membranes and to serve as a key indicator for assessing whether plants are under stress conditions (Gonzalez-Orenga et al., 2023). In comparison with the control group, the MDA content in all stress-treated groups was significantly higher, with a pronounced increase of 2.5- to 3.0-fold in key stress groups. This magnitude of increase signifies substantial membrane damage through lipid oxidation, consistent with the general trend of increased MDA levels in plants subjected to environmental stress under normal conditions. This phenomenon can be attributed to the disruption of the dynamic equilibrium between ROS production and consumption, resulting in elevated intracellular free radical levels and enhanced lipid peroxidation reactions in cell membranes. The end product of this process, known as MDA, commences the process of accumulation (Azeem et al., 2023). The observed fold-change in MDA directly correlates with the degree of membrane integrity loss and cellular dysfunction.

Furthermore, the O_2_•- content in NcL samples increased significantly under stress conditions, with all stress groups exhibiting considerably higher O_2_•- levels than the control group, reaching levels up to 2.0-fold higher than the control. As one of the most fundamental forms of ROS, O_2_•^-^ serves as a crucial signaling molecule within plants, playing a key role in multiple regulatory processes (Karpinska and Foyer, 2024; Kim et al., 2007; Wang et al., 2025). However, its excessive accumulation often indicates that plants are under oxidative stress, triggering a series of adverse reactions within the plant itself. The Fenton reaction or Haber-Weiss reaction, which can generate hydroxyl radicals, is of particular concern. These radicals are extremely reactive and toxic, and are capable of attacking and damaging macromolecules within cells, such as proteins, lipids, and nucleic acids. The result of this is severe oxidative damage (He et al., 2023; Koppenol, 2001). The several-fold increase in O_2_•^-^ underscores its role not only as a signal but also as a primary source of oxidative burden, leading to widespread macromolecular damage.

In summary, the stress conditions disrupted the dynamic equilibrium between ROS production and consumption within NcL, leading to substantial accumulation of MDA and O_2_•- within the plant. This instigated and intensified oxidative damage within cells, primarily through lipid peroxidation (reflected by MDA folds) and radical-mediated attacks (initiated by O_2_•- folds),resulting in impairment to the structure and function of NcL cells. Consequently, the normal physiological processes of the plant were disrupted, including photosynthesis, respiration, water and nutrient transport, and organic compound accumulation, ultimately affecting the plant’s normal growth state (Hua et al., 2020; Jansen et al., 2022; Jia et al., 2025).

Research indicates that stress conditions have a multifaceted impact on plant growth and development, encompassing the domains of plant condition, physiological and biochemical indicators, and oxidative stress markers. A salient finding is the influence of stress conditions on the accumulation of active compounds (Qiuyue et al., 2023; Xiaoqing et al., 2018). Presently, the content of active ingredients in TCM serves as a pivotal indicator of their quality. The analysis of pulegone content in NcL samples, conducted using UPLC, indicates that varying levels of stress significantly influence PG levels, with a substantial decrease observed in samples subjected to specific stress conditions. This finding suggests that salt or heavy metal stress at varying concentrations disrupts the normal structure and function of NcL cells, inducing oxidative stress. This disruption has been shown to impair the plant’s physiological functions, hinder the accumulation of active compounds, and consequently lead to a noticeable decline in the quality of the medicinal material.

As previously stated, the SPE characteristics of living organisms and their representative parameter CPS are closely related to their internal redox metabolic activities. In contrast, their DL characteristics and the parameter I_0_ are closely associated with the process of biomolecules within living organisms transitioning from high-energy states to low-energy states. Changes in the content and chemical composition of active components within NcL affect its redox activities. This, in turn, has a significant impact on the internal energy metabolism of the plant, and consequently on its SPE characteristics. Conversely, the DL characteristics of NcL are contingent on the absorption of external excitation energy by its internal molecules. Alterations in the content and chemical composition of its active components have the capacity to modify the molecular structure within, consequently impacting the absorption and storage capacity of external excitation energy by its biomolecules. This, in turn, impacts the quantity of molecules in high-energy states, influencing the transition process of high-energy biomolecules to low-energy states, affecting biophoton generation, and ultimately affecting its DL characteristics (Gang et al., 2015).

The results of the correlation analysis indicate that under different concentrations and stress conditions, the bioluminescence parameters CPS and I_0_ of NcL exhibit a high degree of correlation with its physiological and biochemical indicators, oxidative stress indicators, and PG content. In the context of varying concentrations and stress conditions, a significant positive correlation was observed between the bioluminescence parameters CPS and I_0_ of NcL and its PG content. Among these parameters, I_0_ exhibited a more pronounced correlation with PG content. Consequently, the levels of bioluminescence parameters CPS and I_0_ can, to a certain extent, reflect the content of its internal active components.

Moreover, in comparison with the control group, the photosynthetic pigment and PG content in NcL samples exposed to diverse stress conditions was found to be considerably diminished overall. The mechanistic link between these observations and the biophotonic parameters can be interpreted as follows. Firstly, the degradation of photosynthetic pigments directly reduces the photon absorption capacity and the population of electronically excited states within the photosynthetic apparatus. Since DL originates from the recombination of charge pairs and the relaxation of excited states in photosystem II, a smaller pigment pool leads to a lower probability of such photo-physical events, thereby directly explaining the suppression of I_0_. Secondly, oxidative stress instigates a dual effect on biophoton emission. On one hand, ROS, particularly O_2_•- and its derivative radicals, and excited carbonyls formed during lipid peroxidation, can themselves be direct sources of photon emission upon their decay, potentially contributing to SPE. On the other hand, chronic and severe oxidative stress leads to the destruction of reaction centers, the dismantling of light-harvesting complexes, and a general collapse of the cellular energy budget. This systemic failure drastically reduces the total number of biomolecules capable of being excited or sustaining redox reactions that generate excited states. Consequently, the overall intensity of both SPE (reflected by CPS), as a measure of the global metabolic redox activity, and DL (reflected by I_0_ and T), as a measure of the photosynthetic system’s capacity to store and release light energy, is profoundly diminished. Future studies employing simultaneous measurements of photon emission alongside specific enzymatic activities (e.g., NADPH oxidase) could more precisely pinpoint the origins of these biophotonic signals. Therefore, the observed decline in biophoton emission under stress is not merely a consequence of suppressed metabolism but a direct physical manifestation of a compromised photophysical and redox environment within the plant cells.

The results of the correlation analysis indicate that CPS and I_0_ exhibit significant positive correlations with chlorophyll, carotenoid, and total photosynthetic pigment content at the overall level, while showing significant negative correlations with REC, ROS production rate, MDA, and O_2_•- content. Consequently, it can be deduced that the bioluminescence parameters CPS and I_0_ can, to a certain extent, reflect the physiological and oxidative stress status of NcL, indicating the integrity of its internal structure and function. However, it is important to note that these correlations, while strong, do not establish causation. To further validate the predictive power of these biophotonic parameters and elucidate the underlying causal pathways, future studies would benefit from employing more advanced statistical methods, such as structural equation modeling (path analysis) or multiple regression models, with larger and more diverse datasets.

The significant correlations between biophotonic parameters (CPS and I_0_) and key physiological, oxidative stress, and PG content metrics provide a scientific basis for the non-destructive assessment of medicinal plant quality. The transferability of this approach appears promising, particularly for other medicinal plants within the Lamiaceae family, such as Salvia spp. and Mentha spp., which share similar photosynthetic characteristics and secondary metabolic pathways. Future research should focus on several key directions: first, developing portable and miniaturized biophoton detection tools to facilitate the translation of this technology from the laboratory to field applications; second, establishing comprehensive biophoton databases based on existing technical and detection frameworks; and third, developing accurate predictive models based on these databases, which is a long-term goal that will require substantial data accumulation and interdisciplinary cooperation to fully realize the potential of biophoton technology in non-invasively predicting and monitoring the accumulation of secondary metabolites (active components). Such developments could pave the way for real-time, non-destructive quality monitoring throughout the herbal supply chain, from raw material assessment to final product grading.

## 5 Limitations and future perspectives

This study has some limitations that should be considered. The experiments were conducted under controlled conditions with a limited sample size, which may affect the generalizability of the findings. Furthermore, the model requires validation across a wider range of environmental conditions and plant genetic backgrounds. Future work should therefore focus on: (1) validating these biophotonic parameters in field or greenhouse production systems with larger sample sizes and more replication cycles; (2) exploring the integration of machine learning models to improve prediction accuracy for complex traits; and (3) testing the transferability of the approach to other economically important medicinal plants.

## 6 Conclusion

This study demonstrates that biophoton parameters, specifically the initial intensity of DL (I_0_), serve as a highly effective non-destructive predictor for the quality of fresh NcL. The key quantitative outcomes reveal a strong positive correlation between I_0_ and the content of the critical quality marker PG, establishing I_0_ as a superior indicator compared to other measured parameters. Furthermore, both I_0_ and CPS of SPE exhibited significant correlations with the plant’s physiological status: they were positively correlated with photosynthetic pigment content (e.g., total chlorophyll) and negatively correlated with oxidative stress markers (e.g., MDA and ROS production rate). These robust correlations confirm that biophoton emission accurately reflects the internal metabolic and oxidative state of NcL under abiotic stress. Consequently, biophoton technology, particularly through the parameter I_0_, presents a powerful and practical tool for the real-time, non-destructive quality assessment of medicinal plants during cultivation, offering a significant advancement towards the modernization of TCM quality control.

## Data Availability

The raw data supporting the conclusions of this article will be made available by the authors, without undue reservation.
