# Supplementary material for: Differential concentrations of NaCl and K2Cr2O7 stress conditions: biophoton characteristics and quality prediction feasibility analysis of fresh Nepeta cataria L. leaves
Source: Front Plant Sci. 2025 Nov 12;16:1714452. doi: 10.3389/fpls.2025.1714452 (PMC12648164; doi:10.3389/fpls.2025.1714452)
Supplement: Supplementary file 1 [file DataSheet1.pdf]

Supplementary Materials

S1 SPE and DL Formulas and Definitions

1. Spontaneous Photon Emission (SPE) Parameter:

CPS (Counts Per Second) was calculated as follows:

CPS is a characterization index of SPE properties, and its calculation formula is as follows(Cao et al., 2023)：

$$CPS = N - n \quad (1)$$

Where:

N is the average photon count per second during sample measurement;

n is the average dark count per second (background noise).

2. Delayed Luminescence (DL) Parameters:

The decay kinetics of DL were analyzed using the Gu parametric model. I_0 and T are characterization metrics for the DL properties, the calculation of which is outlined below: (Cao et al., 2023; Xiaolei, 2014)：

$$I_t = A\mathrm{csch}^2(t/B + C) \quad (2)$$

$$I_0 = A\mathrm{csch}^2 C \quad (3)$$

Where:

I_t is the instantaneous light intensity at time t;

I_0 is the initial intensity at t=0;

B is the sample-specific time constant;

C is a parameter characterizing the initial luminescence intensity characteristics.

The decay time T was defined as the time point at which the light intensity decays to 1/3 of the initial value I_0 (i.e., m=3 was used in this study):

$$T = B\left\{\ln\left(\sqrt{m}\sinh C + \sqrt{(m\sinh^2 C + 1)}\right) - C\right\} \quad (4)$$

S1.2 Parameter Interpretation

The Gu parametric formula (Fm 2) can be fitted nonlinearly to obtain three characteristic parameters: A, B, and C. Parameter A is the system response factor, the value of which is affected by multiple factors, including sample characteristics, the structure of the detection device, and illumination conditions. Parameter B is the sample-specific time constant, which reflects the sample's own time-response characteristics, and parameter C characterizes the initial luminescence intensity characteristics of the sample(Popp et al., 1988; Gu. Qiao, 2012). In the kinetic analysis, It denotes the instantaneous light intensity at a given moment in time.

The time point at which the light intensity decays to 1/m of the initial value I_0 (m = [2, 4]; i.e., m = 2,3 or 4) is recorded as T. In this study, m = 3 is used as the calculation standard.

S2 Photosynthetic pigments Formulas

$$C_a = 9.99 \times A_{665}\text{ nm} - 0.0872 \times A_{642}\text{ nm} \quad (5)$$

$$C_b = 17.7 \times A_{642}\text{ nm} - 3.04 \times A_{665}\text{ nm} \quad (6)$$

$$C_{a+b} = C_a + C_b \quad (7)$$

$$C_c = 4.92 \times A_{474}\text{ nm} - 0.0255 \times C_a - 0.225 \times C_b \quad (8)$$

$$M = C \times \frac{V}{g} \quad (9)$$

Where:

C_a is the mass concentration of chlorophyll a (mg/L);

C_b is the mass concentration of chlorophyll b (mg/L);

C_{a+b} is the mass concentration of total chlorophyll (mg/L);

C_c is the mass concentration of carotenoids (mg/L);

M is the pigment mass content in the sample (mg/g);

V is the volume of the extract (L);

g is the mass of the sample (g).

S3 REC Formulas

$$E = \frac{E_2 - E_1}{E_3 - E_1} \times 100\% \quad (10)$$

The conductivity was measured using a DDS-11A conductivity meter, and the REC was calculated from the following measurements:

E1 is the conductivity of the double-distilled water blank;

E2 is the conductivity of the sample extract after shaking;

E3 is the conductivity of the sample extract after boiling in a 100° C water bath for 30 minutes and cooling to room temperature.

S4 ROS Formulas

$$V = \frac{10 \times (K_1 - K_2)}{W} \quad (11)$$

Where：

k is the slope of fluorescence intensity change over time obtained by linear regression;

K1 is the fluorescence intensity change slope of the experimental group;

K2 is the fluorescence intensity change slope of the blank control group.

S5 MDA Formulas

$$\Delta A532 = A532_m - A532_b \quad (12)$$

$$\Delta A600 = A600_m - A600_b \quad (13)$$

$$\Delta A = \Delta A532 - \Delta A600 \quad (14)$$

$$\text{MDA content} = 53.763 \times \Delta A \div W \quad (15)$$

Where:

A532b is the absorbance of the blank tube at 532 nm;

A532m is the absorbance of the measurement tube at 532 nm;

A600b is the absorbance of the blank tube at 600 nm;

A600m is the absorbance of the measurement tube at 600 nm;

W is the mass of the fresh leaf sample (g).

S6 O_2•- Formulas

$$\Delta A_s = A_s - A_b \quad (16)$$

$$\Delta A_m = A_m - A_b \quad (17)$$

$$O_2\bullet\text{- content} = 0.0625 \times \Delta A_m \div \Delta A_s \div W \quad (18)$$

Where:

Ab is the absorbance of the blank tube at 530 nm;

Am is the absorbance of the measurement tube at 530 nm;

As is the absorbance of the standard tube at 530 nm;

W is the mass of the fresh leaf sample (g).

Reference

Cao, B., Wang, Z., Zhang, J., Fu, J., Zhang, Z., Du, J., . . . Han, J. (2023). A biophoton method for identifying the quality states of fresh Chinese herbs. *Front Pharmacol, 14*, 1140117. doi:10.3389/fphar.2023.1140117

Xiaolei, Z. (2014). *The preliminary study on characteristics of biophoton emission of Chinese herbs in the process of growth* (Thesis).

Popp, F. A., Li, K. H., Mei, W. P., Galle, M., & Neurohr, R. (1988). Physical aspects of biophotons. Experientia, 44(7), 576-585. doi:10.1007/BF01953305

Qiao, G. (2012). *Biophotonics* (2 ed.): Beijing Science Press.

Qiuchan, H. Y., Xu.; Youhuan, Wei.; Yunzhen, Liang. (2013). Comparative Analysis of Leaf Carotenoids Content of the 4 Common Dark Blade Plants. Hubei Agricultural Sciences, 52(11), 2573-2574. doi:10.14088/j.cnki.issn0439-8114.2013.11.060
