# Supplementary material for: Differential concentrations of NaCl and K2Cr2O7 stress conditions: biophoton characteristics and quality prediction feasibility analysis of fresh Nepeta cataria L. leaves
Source: Front Plant Sci. 2025 Nov 12;16:1714452. doi: 10.3389/fpls.2025.1714452 (PMC12648164; doi:10.3389/fpls.2025.1714452)
Supplement: Supplementary file 2 [file DataSheet2.pdf]

Supplementary Table. Mean and standard deviation (SD) values for the data presented in Figures 4-13.

Table. 1 Mean and SD values for the data presented in Figure 4

Figure	Panel	Group	Stage	Mean	SD
4	a	Control	1	115.024	3.274
			2	86.394	2.691
			3	90.424	4.284
			4	89.164	3.771
		NaCl 60mmol/L	1	120.785	4.551
			2	94.331	3.034
			3	56.426	4.224
			4	63.803	4.551
		NaCl 120mmol/L	1	112.305	3.837
			2	63.328	2.812
			3	32.859	3.563
			4	40.767	2.254
	b	Control	1	2.387×10^{6}	6.506×10^{4}
			2	2.257×10^{6}	6.028×10^{4}
			3	1.926×10^{6}	6.028×10^{4}
			4	1.137×10^{6}	8.607×10^{4}
		NaCl 60mmol/L	1	2.110×10^{6}	1.205×10^{5}
			2	2.050×10^{6}	7.000×10^{4}
			3	9.093×10^{5}	2.003×10^{4}
			4	7.823×10^{5}	2.861×10^{4}
		NaCl 120mmol/L	1	2.173×10^{6}	6.506×10^{4}
			2	8.500×10^{5}	8.000×10^{4}
			3	5.523×10^{5}	1.923×10^{4}

			**4**	5.217×10^5	4.752×10^4
	**c**	**Control**	**1**	3.097	0.031
			**2**	3.238	0.027
			**3**	3.375	0.020
			**4**	3.497	0.029
		**NaCl 60mmol/L**	**1**	3.410	0.033
			**2**	3.152	0.037
			**3**	3.596	0.044
			**4**	3.017	0.019
		**NaCl 120mmol/L**	**1**	3.999	0.047
			**2**	3.209	0.027
			**3**	3.355	0.057
			**4**	3.313	0.044

Table. 2 Mean and SD values for the data presented in Figure 5

Figure	Panel	Group	Mean	SD
**5**	**a**	**Control**	89.16	3.770
		**NaCl 60mmol/L**	63.80	4.552
		**NaCl 120mmol/L**	40.77	2.760
	**b**	**Control**	1.137×10^6	8.737×10^4
		**NaCl 60mmol/L**	7.823×10^5	2.857×10^4
		**NaCl 120mmol/L**	5.217×10^5	4.752×10^4
	**c**	**Control**	3.497	0.02882
		**NaCl 60mmol/L**	3.017	0.01907
		**NaCl 120mmol/L**	3.313	0.04487

Table. 3 Mean and SD values for the data presented in Figure 6

Figure	Panel	Group	Stage	Mean	SD
6	a	Control	1	115.024	3.274
			2	86.394	2.691
			3	90.424	4.284
			4	89.164	3.771
		$K_2Cr_2O_7$ 15mg/L	1	117.158	3.455
			2	90.131	2.997
			3	70.998	2.345
			4	41.152	3.229
		$K_2Cr_2O_7$ 45mg/L	1	119.222	3.035
			2	93.063	3.587
			3	60.232	2.966
			4	30.965	3.037
	b	Control	1	2.387×10^6	6.506×10^4
			2	2.257×10^6	6.028×10^4
			3	1.926×10^6	6.028×10^4
			4	1.137×10^6	8.607×10^4
		$K_2Cr_2O_7$ 15mg/L	1	2.363×10^6	3.041×10^4
			2	1.700×10^6	5.568×10^4
			3	1.327×10^6	3.693×10^4
			4	7.917×10^5	5.066×10^3
		$K_2Cr_2O_7$ 45mg/L	1	2.290×10^6 ;	4.359×10^4
			2	1.610×10^6	3.606×10^4
			3	6.850×10^5	1.670×10^4
			4	5.193×10^5	2.710×10^4
	c	Control	1	3.097	0.031
			2	3.238	0.027
			3	3.375	0.020

			**4**	3.497	0.029
		**$K_2Cr_2O_7$ 15mg/L**	**1**	3.087	0.025
			**2**	3.869	0.040
			**3**	3.390	0.029
			**4**	4.100	0.026
		**$K_2Cr_2O_7$ 45mg/L**	**1**	3.100	0.040
			**2**	3.804	0.038
			**3**	3.311	0.046
			**4**	3.551	0.030

Table.4 Mean and SD values for the data presented in Figure 7

**Figure**	**Panel**	**Group**	**Mean**	**SD**
**7**	**a**	**Control**	89.160	3.770
		**$K_2Cr_2O_7$ 15mg/L**	41.150	3.229
		**$K_2Cr_2O_7$ 45mg/L**	30.960	3.107
	**b**	**Control**	1.137×10^6	8.737×10^4
		**$K_2Cr_2O_7$ 15mg/L**	7.917×10^5	5.132×10^3
		**$K_2Cr_2O_7$ 45mg/L**	5.193×10^5	2.710×10^4
	**c**	**Control**	3.497	0.029
		**$K_2Cr_2O_7$ 15mg/L**	4.100	0.028
		**$K_2Cr_2O_7$ 45mg/L**	3.551	0.030

Table.5 Mean and SD values for the data presented in Figure 8

Figure	Group	Group	Mean	SD
8	TPP	Control	10.353	0.245
		NaCl 60mmol/L	4.037	0.180
		NaCl 120mmol/L	6.693	0.059
		$K_2Cr_2O_7$ 15mg/L	8.877	0.180
		$K_2Cr_2O_7$ 45mg/L	3.510	0.193
	Chlorophyll	Control	8.107	0.191
		NaCl 60mmol/L	3.267	0.143
		NaCl 120mmol/L	5.370	0.035
		$K_2Cr_2O_7$ 15mg/L	6.337	0.101
		$K_2Cr_2O_7$ 45mg/L	2.750	0.143
	Carotenoid	Control	2.280	0.112
		NaCl 60mmol/L	0.770	0.043
		NaCl 120mmol/L	1.323	0.025
		$K_2Cr_2O_7$ 15mg/L	2.540	0.082
		$K_2Cr_2O_7$ 45mg/L	0.760	0.052

Table.6 Mean and SD values for the data presented in Figure 9

Figure	Group	Mean	SD
9	Control	0.330	0.011
	NaCl 60mmol/L	0.418	0.015
	NaCl 120mmol/L	0.428	0.004
	$K_2Cr_2O_7$ 15mg/L	0.234	0.009
	$K_2Cr_2O_7$ 45mg/L	0.686	0.065

Table.7 Mean and SD values for the data presented in Figure 10

Figure	Group	Mean	SD
10	Control	2.888×10^{8}	2.387×10^{6}
	NaCl 60mmol/L	1.054×10^{9}	3.362×10^{7}
	NaCl 120mmol/L	1.186×10^{9}	4.393×10^{7}
	$K_2Cr_2O_7$ 15mg/L	4.920×10^{8}	2.566×10^{7}
	$K_2Cr_2O_7$ 45mg/L	1.198×10^{9}	9.757×10^{7}

Table.8 Mean and SD values for the data presented in Figure 11

Figure	Group	Mean	SD
11	Control	22.20	1.440
	NaCl 60mmol/L	36.79	1.346
	NaCl 120mmol/L	52.49	0.7948
	$K_2Cr_2O_7$ 15mg/L	31.95	1.710
	$K_2Cr_2O_7$ 45mg/L	40.50	2.172

Table.9 Mean and SD values for the data presented in Figure 12

Figure	Group	Mean	SD
12	Control	0.258	0.007
	NaCl 60mmol/L	0.312	0.012
	NaCl 120mmol/L	0.827	0.021
	$K_2Cr_2O_7$ 15mg/L	0.323	0.020
	$K_2Cr_2O_7$ 45mg/L	0.417	0.014

Table.10 Mean and SD values for the data presented in Figure 13

Figure	Group	Mean	SD
13	Control	2.038	0.062
	NaCl 60mmol/L	1.533	0.106
	NaCl 120mmol/L	1.219	0.089
	$K_2Cr_2O_7$ 15mg/L	1.586	0.032
	$K_2Cr_2O_7$ 45mg/L	1.175	0.061
